# Reorganization of work schedules for better distribution of work demands in home health care – a feasibility study

**DOI:** 10.1186/s12913-025-12746-1

**Published:** 2025-04-26

**Authors:** Maja Vilhelmsen, Tonje Pedersen Ludvigsen, Kristina Thomassen, Charlotte Diana Nørregaard Rasmussen

**Affiliations:** 1 National Research Centre for the Working Environment, Copenhagen, Denmark; 2 Center for Clinical Research and Prevention, Frederiksberg, Denmark

**Keywords:** Goldilocks work principle, Organizational intervention, Home health care workers, Home health care institution, Fidelity, Psychosocial work demands, Physical work demands, Participatory intervention

## Abstract

**Background:**

Given the home health care industry’s growth, increasing demand for workers, and complex patient care needs, investigating initiatives to maintain home health care workers’ health and ability to work is crucial. This study aims to assess the feasibility of an organizational intervention for equal distribution of physical and psychosocial work demands among home health care aides.

**Method:**

We conducted a 7-week quasi-experimental feasibility study at a Danish home health care institution with 27 home health care aides. The 6SQuID framework was used to develop, test and evaluate the feasibility of the organizational intervention, inspired by the ‘Goldilocks Work Principle’. The intervention consisted of three activities: (1) classification workshop, (2) individual dialogue with a schedule coordinator, and (3) reorganizing work schedules. Feasibility was assessed through: (1) acceptability evaluated by interview and questionnaire post-intervention, (2) fidelity assessed by documentation during intervention, and (3) potential effects on selected psychosocial factors and physical work demands evaluated pre-post intervention with technical measurements and questionnaire.

**Results:**

Nineteen home health care aides participated in the evaluation of the intervention. Most of the home health care aides (73.33%) reported to like or really like the intervention. The interviewees expressed general acceptance of both the intervention activities and the overall aim of the intervention. Most home health care aides (77.8%) participated in the Classification workshop and 124 citizens were classified. All home health care aides participated in the Individual dialogue. No significant changes were seen in the Reorganized work schedules (*p* > 0.05). Physical and emotional fatigue and physical exertion showed statistically significant change (*p* < 0.05), with a mean difference of 17 and 11 (100 point scale), and 1.7 (10 point scale) points respectively.

**Conclusion:**

This study found components of the intervention to be feasible, but concludes that adaptions to enhance implementation addressing barriers related to time pressure, improving fidelity to the intervention, and ensuring practical applicability within the home health care context are critical for future success.

**Trial registration:**

The study was registered in the ISRCTN registry under registration number ISRCTN15131198 on August 8, 2023.

**Supplementary Information:**

The online version contains supplementary material available at 10.1186/s12913-025-12746-1.

## Background

An aging population, with more people living with chronic conditions, has required improvements in technology and medical advances, enabling the provision of more complex patient care in the patient’s own home. This results in a steadily growing home healthcare (HHC) industry, and the need for more HHC-workers [1]. Therefore, to keep up with the increasing need for care, initiatives for keeping the HHC-workers healthy and working should be investigated.

In Denmark, HHC-aides are one of three main professions working in HHC industry together with HHC-assistants, and nurses. The HHC-aides undergo a comparatively short education (14 months of training) and differ from HHC-assistants and nurses in two important ways; the amount of time they spend with each citizen, at least one hour, and the broad spectrum of tasks during patient visits, including direct care as well as indirect care such as laundry, cleaning, and cooking [2, 3]. Thus, HHC-aides have the most prominent physical exposures, such as highly repetitive work, heavy lifting/pushing/pulling and prolonged standing [4]. A large proportion of the physical and psychosocial demands in homecare work stems from the interaction and care for the citizens. Exposure to high physical and psychosocial demands challenges a healthy and capable workforce [5], due to increased risk for musculoskeletal pain and burnout [6], leading to impaired work ability and long-term sickness absence [3, 7, 8].

The amount of aid and support provided during each home visit depends on the individual needs of the citizen, determined by the local authority [9]. Consequently, the physical and psychosocial work demands vary from one visit to another. A designated scheduling coordinator (SC) is typically responsible for managing the distribution of visits among HHC-aides, considering factors such as the relationship between HHC-aides’ and citizens, time-sensitive appointments, and daily changes arising from the evolving care situations of the citizens [10]. However, there seems to be a lack of systematic approach for the consideration of the physical and psychosocial demands when planning and distributing visits among HHC-aides. This is indicated by a study conducted in Norway, that used wearable-based measurements to assess physical work demands across 13 HHC-units, showing an uneven distribution of work tasks among the HHC-workers [11]. Furthermore, the physical effort required to care for a citizen have shown to be an important predictor for end-of-day pain [2] and physical exertion [12]. Thus, knowledge about how to plan and organize the visits to reduce high work demands for HHC-aides is needed.

In recent years, the ‘Goldilocks Work Principle’ has been proposed as an approach to designing productive work that promotes health and physical capacity by finding the ‘just right’ distribution of physical demanding work tasks [13]. The approach has been tested and shown to be feasible in an industrial setting where participating individuals experienced less fatigue, reduced pain and higher energy levels than the control group [14]. Additionally, a study conducted in nursing homes found a significant correlation between fairer task distribution and lower perceived physical exertion among health care workers, highlighting the importance of equal work task distribution [4]. Furthermore, two studies of relevance have been published in recent years, one feasibility study [2] and one protocol article [15], targeting the distribution of physical work demands among HHC-workers. However, to our knowledge, no study has addressed how to organize work in a way that accounts for the distribution of physical and psychosocial work demands when planning work schedules.

The aim of this study is therefore to assess the feasibility of an organizational intervention designed to achieve a more equal distribution of physical and psychosocial work demands among HHC-aides in a Danish municipal using a participatory approach to adapt the intervention to the specific context. Feasibility was assessed through three key dimensions, in line with recommendations by Bowen et al. [16]: Acceptability, Fidelity (implementation), and potential effects (Limited Efficacy). Under *acceptability*, we investigated the acceptance of the intervention among HHC-aides. Under *fidelity*, we evaluated the degree to which the intervention was implemented as intended. Under *potential effects*, we examined the anticipated outcomes on selected psychosocial and physical work demands to provide insight into the intervention’s potential effect on health and the work environment.

## Method

### Study design and recruitment

This study was designed as a pretest-posttest quasi-experimental feasibility study, conducted between January and December 2022. Participating HHC-aides were recruited in an HHC-unit, employing 27 HHC-aides, and based in a Danish Municipality (Frederiksberg). In this unit, the HHC-visits are distributed among the HHC-workers by two SC, with one being responsible for the HHC-aides’ work schedules and the other being responsible for the HHC-assistances’ and nurses’ work schedule. The HHC-aides were further divided into two team, based on geographic location of the HHC-visits. All HHC-workers walked or used bicycles for transportation between HHC-visits. Since this study is an organizational work environment intervention, all HHC-aides were expected to participate in the intervention and were eligible to participate in the evaluation of the intervention.

### Designing the workplace intervention

We used the 6SQuID (Six Steps for Quality Intervention Development) framework to develop, test and evaluate the feasibility of the intervention [17], which was inspired by the ‘Goldilocks Work Principle’ [13]. Step 1–4 of the framework guided the needs assessment and the co-creation process of the intervention activities with the HHC-institution, while steps 5–6 guided the implementation and evaluation of the intervention.

**Fig. 1:**
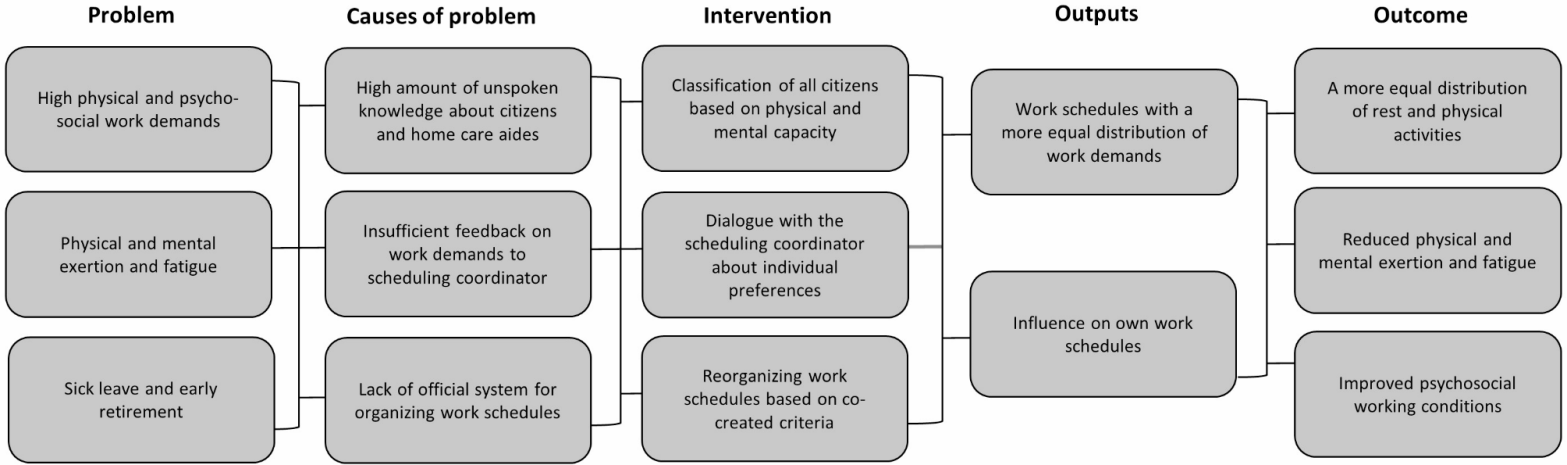
Program logic

The intervention targeted the organization of physical and psychosocial demanding work tasks, as seen in the program logic in Fig. 1. In step one and two, we conducted field visits and informal interviews to gather information about the organization of the workday, factors influencing the planning process, and the digital planning system. We found a lack of official guidelines and an abundance of unspoken knowledge in organizing work schedules. Additionally, we found differences in how HHC-aides provided feedback to the SC, which influenced the distribution of physically and psychosocially demanding work tasks. This information was used in step three, were a work team consisting of six HHC-employees, one team manager, and one internal consultant investigated possible change mechanisms. Two classification scales; ‘Need for Physical Assistance scale [18] and ‘Mental and Social Behavior scale’, were presented to the work team and used to quantify the physical and psychosocial work demands encountered during visits to citizens’ homes and create a common language about them between the SC and the HHC-aides. Each scale is divided into four categories. The Need for Physical Assistance scale is divided into; 1) Light physical assistance, 2) Moderate physical assistance, 3) Extensive physical assistance and 4) Total physical assistance. The Mental and Social Behavior scale is divided into; 1) Neutral behavior, 2) Demanding behavior, 3) Controlling or resisting behavior and 4) Violent behavior. In step four, the work team developed criteria for distributing citizens among HHC-aides based on the citizen’s need for physical assistance and their mental and social behavior. The criteria were designed to guide the SC when planning the workday for each HHC-aide. The intervention activities were implemented and evaluated in a seven-week intervention period (Step 5 and 6).

### Workplace intervention

The workplace intervention consisted of three activities identified in the co-creation process; illustrated in the Program logic in Fig. 1 and described below.

A *Classification Workshop* (30 min), where HHC-aides in groups of four-six classified their primary citizens using the Need for Physical Assistance scale and Mental and Social Behavior scale. Discrepancies were sorted out through group discussion until agreement. All classifications were delivered to SC.

An *Individual Dialogue* (5–10 min) between the SC and each HHC- aide focusing on individual preferences and conditions of the HHC-aides related to the workday (e.g. allergies, injuries or difficult relations to specific citizens). Information was noted in a excel sheet to be used by SC when planning the work schedules.

To *Reorganizing Work Schedules* by using the classification of each citizen and a set of new criteria for the SC to use when planning the workday. The three criteria were; (1) maximum three citizens categorized as 4 (i.e. “Total physical assistance” or “Violent behavior” on the Need for Physical Assistance or Mental and Social Behavior scale respectively), (2) no citizens categorized as 4 consecutively unless there is a lunch break or a meeting in between and (3) all cleaning tasks are classified as a category 4. These criteria had to be met daily for every HHC-aides during weekdays. To ensure this, classifications of all citizens needed to be integrated into the digital program used to organize work schedules.

### Data collection

**Fig. 2:**
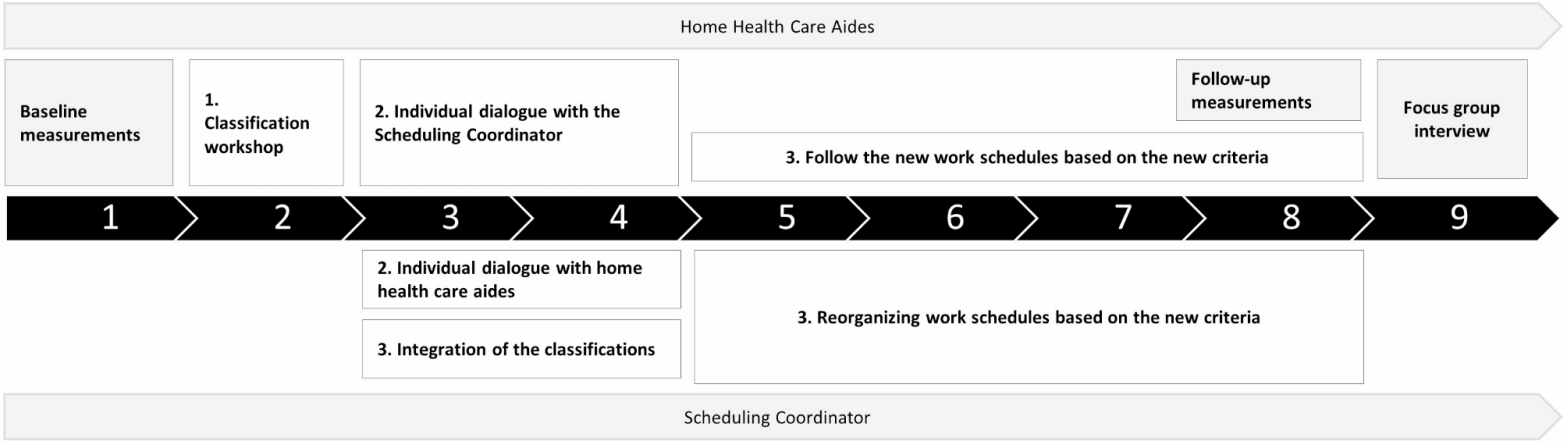
Intervention and evaluation timeline (weeks) for home health care aides (HHC-aides) and scheduling coordinator (SC)

Data was collected before, during and after the intervention with multiple methods including questionnaires, interviews, collection of work schedules and technical measurements of physical behavior as shown in Fig. 2*.* At baseline, characteristics of participants were collected, including questions about sex, seniority, and weekly working hours.

#### Acceptability

The study evaluated the acceptability of the three intervention activities (the classification workshop, the dialogue with SC and the reorganization of work schedules) based on the Theoretical Framework of Acceptability (TFA) [19] using questionnaires and interviews. We assessed acceptability retrospectively (at follow-up) by examining *experienced cognitive and emotional responses to the intervention* of HHC- aides, focusing on the seven constructs of TFA, as shown in Table 1. A validated TFA questionnaire [20] was used to assess five of the seven TFA constructs. It included 13 statements, e.g. "The classification of citizens has improved the distribution of physical and psychosocial demands during the workday" with response options on a Likert scale going from “Completely agree” to “Completely disagree”. The questions are detailed in Table 3, within the Results Section. The construct ‘overall acceptability’ enabled exploration of which constructs drives the general acceptability of the intervention, which we cannot conclude from the sub-constructs alone.

**Table 1:** Overview of how the seven constructs of ‘the theoretical framework of acceptability’ were investigated with either questionnaire, interview, or both

Constructs	Overall acceptability	Affective attitude	Burden	Opportunity cost	Intervention coherence	Ethicality	Perceived effectiveness	Self-efficacy
***Questionnaire***	x	x	x		x		x	
***Group interview***	x	x	x	x	x	x	x	x

**Table 2:** Demographics and work characteristics

**Gender**	**N**	**%**
Female	17	90.0
Male	2	10.0
**Seniority**		
Less than 1 year	1	5.26
1-3 years	3	17.76
4-6 years	4	21.05
7 or more	11	57.89
**Working hours per week**	**Mean **	**SD**
	34.8	2.5

*SD *Standard deviation

To elaborate on the results from the questionnaire, we carried out a semi-structured group interview with four HHC-aides at the HCC-institutions’ facilities. The interview guide was structured around the seven constructs of TFA and lasted approximately one hour, during working hours. The guide included questions such as “*How do you think it has been to be a part of the project?*” (assessing affective attitude), “*What has been required of you as employees to participate in the project?*” (assessing burden), and “*Can you describe some changes that you have observed in your workday because of the project’s activities?*” (assessing effectiveness). Additionally, the guide focused on gathering interviewees’ feedback and suggestions for adjustments to the intervention, with questions like, “*What would need to change for it to require less from you?”* and “*What would you do if you were to plan schedules that create a better distribution of citizens and a greater balance in workload?”.* The detailed interview guide can be found in APPENDIX B. One of the authors (MVI) carried out the interview, while a second observed and took notes (TPL). The interview was recorded with a dictaphone and saved as an audio file.

#### Fidelity of intervention activities

To assess aspects of fidelity [21] we investigated if the intervention activities were delivered and conducted as planned. We registered number of participants at the workshops and number of citizens classified, and number of dialogues conducted between HHC-aides and SC. Further, we collected work schedules in paper form for all HHC-aides two weeks prior to the intervention and during the four weeks of intervention. This enabled a descriptive comparison of the degree to which the work schedules met the criteria set by the work team. The work schedules were typed into a template in Excel.

#### Psychosocial working conditions, fatigue and exertion

Data on psychosocial working conditions, exertion and fatigue was collected through questionnaires at baseline and follow-up. The questionnaire contained questions from validated questionnaires related to: (1) psychosocial working environment, including 13 questions from the Danish Psychosocial Questionnaire regarding four constructs; workload, work pace, emotionally demands (responses on a 5-point Likert scale, where 1 is “never under pressure” and 5 is “always under pressure”) and influence at work (responses on a 5-point Likert scale, where 1 is “low influence” and 5 is “high influence”) [22], (2) Physical and emotionally fatigue (responses on a 5-point Likert scale, where 1 is “never fatigued” and 5 is “always fatigued”) [23], and (3) Physical exertion during work (responses on a 0–10 response scale, where 0 is no exertion and 10 is maximal exertion) [24].

#### Physical behavior

Physical behavior of the participants was measured using a triaxle accelerometer (SENS Motion^®^, Copenhagen, Denmark) attached to their right thigh (midway on the line between the anterior inferior iliac spine and the top of the patella [25]) for 24 h over five consecutive days. Participants logged their work hours, leisure time and sleep using an app. The accelerometer (47 × 22 × 4.5 mm, 7 g) recorded, sampled and stored trial-axial acceleration data at a frequency of 25 Hz with a measurement range of ± 4 g. The data was processed using Acti4, a custom-made MATLAB based software (The National Research Centre for the Working Environment, Copenhagen, Denmark) [25], to determine physical behaviors such as time spent sitting, standing still, standing with movement, walking slow, walking fast, stair climbing, running and cycling. The physical behavior was further categorized into three categories: sedentary behavior (lying and sitting), LPA (standing, move and walking slow), and MVPA (walking fast, stair climbing, running and cycling). Non-movement periods lasting for more than 60 min were considered as non-wear time or sleep. Workdays with less than 4 h of accelerometer recordings were excluded from the analysis.

### Data analysis

#### Interviews– qualitative analysis

Selected parts of the interview were transcribed and analyzed using the software program NVivo (version 11). Our selection was based on relevance according to the constructs in TFA and the transcripts underwent a deductive analysis inspired by Elo & Kyngäs qualitative content analysis process [26]. We developed a coding matrix based on the seven constructs of TFA, to which we applied ‘meaning units’ of the transcripts. The analysis was primarily carried out by one researcher (MVI), supported by two researchers (TPL/KRT). The findings from the interview are presented under the theoretical constructs of TFA together with the results from the questionnaire regarding acceptability.

#### Statistics– quantitative analysis

Data analysis was performed using R Studio (version 1.4.1717) with Tidyverse (version 1.0.10) package. Furthermore, the packages compositions (version 2.0–4), ggplot2 (version 3.4.0) and ggtern (version 3.4.1) were imported allowing generation of ternary plots to show compositional changes of physical behavior for all HHC-aides. Excel was used to calculate the amount of work schedules meeting the criteria on group level. We calculated the number (n) and percentage (%) of work schedules, which met none (0), one (1) or both (2) criteria per week and in total.

Descriptive statistics, such as group means and standard deviations, were used to summarize data collected at both baseline and follow-up. For continuous variables, numerical differences and 95% confidence intervals were used to present differences in outcomes between baseline and follow-up. For questionnaire data related to psychosocial working conditions and fatigue, the Likert scale was transformed into a 100-point scale where 100 represented “To a very high degree,” 75 represented “To a high degree,” 50 represented “To some degree,” 25 represented “To a slight degree,” and 0 represented “Not at all.” A score for each participant for each construct from 0 to 100 was calculated and used to determine the overall mean score of each construct. Parametric and non-parametric test, dependent on the distribution of data was used to present 95% confidence.

### Data protection, ethical evaluation, and trial registration

The National Research Center for the Working Environment has an institutional agreement with the Danish Data Protection Agency about procedures to treat confidential data (journal number 2015 - 41- 4232), such as by securing data on a protected drive with limited access and making all individual data pseudonymous. The Committees on Health Research Ethics for the Capital Region of Denmark has evaluated a description of the study and concluded that, according to Danish law as defined in Committee Act § 2 and § 1, the intervention described did not require formal ethical approval (reference number 18041423). We obtained written, informed consent from all participants before enrollment in the evaluation. All data were stored and analyzed according to the current guidelines for data protection [27]. The study is registered in the ISRCTN Registry (ISRCTN15131198). The CONSORT for Pilot and Feasibility Trials Checklist for reporting of interventions [28] were used to ensure comprehensive reporting (Appendix A).

## Results

### Participants

**Fig. 3:**
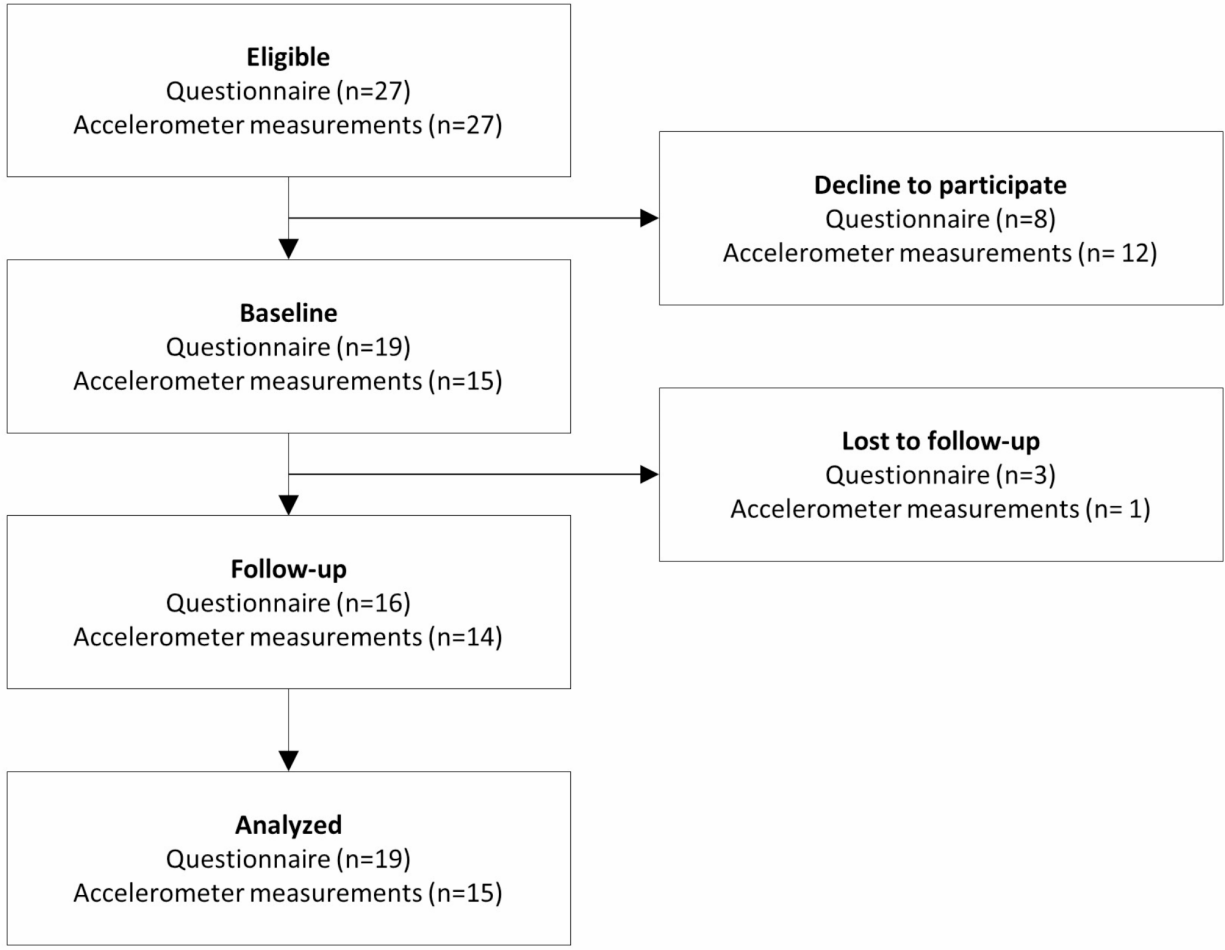
Participants flow for recruitment and participation in the study evaluation

There was 27 HHC-aides eligible for participating in the evaluation of the intervention (Fig. 3), conducted from August to October 2022. Demographics and work characteristics is provided in Table 2. The baseline questionnaire was answered by 19 HHC-aides (70%), yielding a total sample size of 19 HHC-aides. The follow-up questionnaire was sent to participants after the intervention and 15 of the 19 participants (79%) responded. Four female HHC-aides with a seniority between 5 and 14 years participated in the interview.

**Table 3:** Descriptive statistic for the questionnaire data on acceptability at follow-up

**General acceptability**	**Completely ** **agree (%)**	**Agree** **(%)**	**Neutral** **(%)**	**Disagree** **(%)**	**Completely ** **disagree (%)**
I liked the project	26.67	46.67	26.67	0.00	0.00
**Affective attitude**	**Strongly ** **like (%)**	**Like** **(%)**	**Neutral** **(%)**	**Dislike** **(%)**	**Strongly ** **dislike (%)**
What do you think of the new work schedules?	13.33	33.33	33.33	20.00	0.00
What do you think of the individual conversation with the SC*, where you looked at your work schedule together?	13.33	40.00	40.00	6.67	0.00
What do you think about the way citizens were classified?	40.00	40.00	13.33	6.67	0.00
***Burden ***	**No effort** **at all (%)**	**A little ** **effort (%)**	**Neutral** **(%)**	**A lot of** **effort (%)**	**Huge ** **effort (%)**
How much did it require of you to follow the new work schedules?	6.67	46.67	26.67	6.67	0.00
How much did it require of you to classify the citizens?	20.00	66.67	6.67	6.67	0.00
**Intervention coherence**	**Completely ** **agree (%)**	**Agree** **(%)**	**Neutral** **(%)**	**Disagree** **(%)**	**Completely ** **disagree (%)**
*It is clear to me:*..how the new work schedules have contributed to a more equal distribution of physical and psychosocial demands	6.67	66.67	26.67	0.00	0.00
..how the conversation with the SC has contribute to a more equal distribution of physical and psychosocial demands	13.33	60.00	20.00	6.67	0.00
..how the classification of citizens has contributed to a more equal distribution of physical and psychosocial demands	26.67	46.67	20.00	6.67	0.00
**Perceived effectiveness**	**Completely** ** agree (%)**	**Agree** **(%)**	**Neutral** **(%)**	**Disagree** **(%)**	**Completely** ** disagree (%)**
The new work schedules have improved the distribution of physical and psychosocial demands during the workday	0.00	46.67	40.00	13.33	0.00
The conversation with the SC has improved the distribution of physical and psychosocial demands during the workday	0.00	46.67	40.00	13.33	0.00
The classification of citizens has improved the distribution of physical and psychosocial demands during the workday	0.00	46.67	40.00	13.33	0.00

*SC Scheduling coordinator

### Acceptability

The following results present participants’ acceptance of the intervention. All questionnaire results are reported first in Table 3, followed by five paragraphs organized according to seven key constructs of TFA, emphasizing selected questionnaire results together with the findings from the interview. Due to the interrelated nature of the findings in some constructs, we have grouped certain constructs together in the presentation.

#### General acceptability

As seen in Table 3, answers from the follow-up questionnaire showed that most of the HHC-aides (73.33%) reported to like or really like the intervention. The interviewees expressed general acceptance of both the intervention activities and the overall aim of the intervention. They highlighted that “*it would be nice if it (work demands) were more equally distributed*” as that would contribute to work schedules that are “*not just divided into a ‘tough/heavy’ schedule and a ‘coffee’ schedule”.*

#### Affective attitude

Around half of HHC-aides (46.67%) reported in the questionnaire that they liked or very much liked the *reorganized work schedules*, whereas one-fifth (20%) did not like them. The majority (73.33%) reported that the purpose of reorganizing work schedules was clear. Interviewees expressed that it had been fine to participate in the intervention but were unsatisfied that the criteria had not been applied during the weekends. Further, one of them found that the intervention had led to a strong focus on category 4 citizens (the most demanding citizens):

*I think that there are some who are concerned if a citizen is a 4. But it is just as important that you don’t only have category 1 citizens*,* because then you might have 14 visits*,* because they ‘only’ are a category one. And that is just as ‘heavy’ as having category four.*

Half of HHC-aides (53.33%) reported to either like or very much like the dialogue with SC. The interviewees expressed that the dialogue addressed the number of ‘demanding’ citizens on their usual work schedule and homes where they felt unwelcome. However, they found the timeframe (5–10 min) was too short to address this. Further, they missed a clearer purpose and structure and emphasized that the dialogue *“needs to be prepared so the scheduling coordinator knows what to talk about*”. One interviewee suggested that the SC used the dialogues to make an overview of preferences and conditions of HHC-aides, e.g. relevant allergies and phobias, as a way to systematically register and use the knowledge gained in the dialogue.

Almost all (80%) HHC-aides reported to like *the classification of citizens*. This was also the case for the interviewees, but one found the classification workshop “*hurried*” as the timeframe (20 min) was too short. Another interviewee described that classifying in groups revealed differences between colleagues in the judgement of the physical and/or mental state of a citizen, e.g., when discussing the category of a citizen:

”*There was a difference in what we thought in the team. Sometimes*,* I was like ‘Okay*,* this citizen is a category one’ and then my colleague would argue ‘No*,* that citizen is definitely a category three’. And I was just thinking ‘Okay*,* why would you think that? I think it is a category one.”*

#### Burden and opportunity cost

The majority of HHC-aides reported that *the classification of citizens* required nothing (20.00%) or little (66.67%) of them, and half of them (53.33%) reported that it required no or little effort to follow the reorganized work schedules, while 20% reported that it took a lot or huge effort to follow reorganized work schedules. Statements of the interviewees supports these findings, as they described that they did not have to give up opportunities or make compromises, e.g. on quality of work or continuity, during the intervention period. However, one pointed out that continuous classification of e.g. of new citizens during the daily lunch meeting, could result in accelerated work pace, as HHC-aides would have to ‘*save up extra time’* during the workday:

*We have to find that extra time to be able to actually join that meeting. It is not something we have time to do. We have to take it from our visits to all the citizens*,* so it could be very stressful*

Another interviewee agreed calling it “*extra unnecessary work*”.

#### Self-sefficacy and ethicality

All interviewees expressed that they felt capable in classifying citizens. However, one told that she felt in doubt about the accuracy afterwards and got *“a bit uncertain”*. In contrast, another interviewee expressed doubt about their colleagues’ ability to classify correctly:

*“I think some of them (the colleagues) did not exactly understand. like*,* the purpose. Because in the beginning they all thought that all the citizens were a category 4 (.) where I was like ‘everybody is not just a category 4’”.*

Furthermore, the interviewees were uncertain of the capability and efficacy of the SC to take work demands into account and meet the new criteria when organizing work schedules, especially in periods with sick leave or vacation, where there is a lack of employees. Because, as one interviewee said, in these periods *“the pieces of the puzzle have to fit”.*

The interviewees did not report any ethical or value related problems with the intervention. Instead, they told, that preferences of citizens, according to them, were given too much attention in the organization of work schedules compared to the physical and psychosocial demands of the schedules:

*“I sometimes think that the citizens have too big influence on who is visiting them (.) Like ‘I don’t want this person*,* and I don’t want this person*,* I just want her or him'. Where I just think*,* you can’t just decide that”.*

Hence, interviewees found the intervention’s stronger focus on their work demands and work environment valuable and relevant.

#### Intervention coherence and perceived effectiveness

The majority of HHC-aides agreed (60%) or completely agreed (13.33%) that they understood how the classification contributed to a more equal distribution of work demands. This was confirmed by the interviewees, who described the purpose of the intervention several times as achieving “*some sort of equal distribution of ‘easy’ and ‘demanding’ citizens*”, and further emphasized that *“it would be ideal if everybody had that”*. Furthermore, interviewees were aware of the complexity of reorganizing work schedules in a new way for the SC, taking several factors, including work demands, into account:

*But it is also difficult for the one who organizes the work schedules*,* because then this HHC-aides cannot come in this home because of smoke*,* or because the citizen doesn’t like the HHC-aide*,* or because the HHC-aide is a foreigner (.) So there is a lot of things to take into account*

As a result of this complexity, interviewees described it as ‘*difficult*’ to follow the criteria and influence the distribution of work demands. In the questionnaire, just below half of HHC-aides agreed (46.67%) that the intervention contributed to a more equal distribution of physical and psychosocial work demands, whereas 13.33% disagreed. Interviewees described that they did not experience an effect of the classification, the dialogue, or the reorganized work schedules. One stated “*I don’t think there is a big difference*”, and described still having “*the coffee list*,* as we call it*”, one week and then “*the really heavy schedule*” the next week, concluding “*something is not working here*”. However, this was during the weekend when the work schedules had not been reorganized. According to interviewees, a reason for the difficulty fulfilling the criteria could be that some classifications were not typed into the digital system yet, meaning that the SC could not take them into account when organizing work schedules. Further, they did not experience classifying any new citizens during the intervention period or re-classifying citizens due to change in physical or mental state.

### Fidelity

Twenty-one out of all the HHC-aides (77.8%) participated in the classification workshop and 95 citizens were classified. Reasons for not participating were vacation or sick leave. During the first week of the intervention, four employees classified an additional 74 citizens. This was due to elimination of criterion 3; all cleaning tasks are classified as a category 4, as it was unfeasible. In total, 169 citizens were classified by the HHC-aides. The SC reported conducting individual dialogues with all 27 HHC-aides individually and creating an Excel document to register their work preferences.

Before the intervention, 141 work schedules (70 per week) were planned during two weeks, compared to 316 (79 per week) during the four-week intervention period. During both periods, the daily number of planned visits at a category 4 citizen divided by the total number of HHC-aides working that day was 2.62 visits (range 2.4–3.0) and 2.57 visits (range 2.3–2.9), respectively. Thus, the average amount of category 4 visits per day was under three qualifying criteria 1 (maximum three category 4 visits per day) to be feasible.

Table 4 presents the percentage of work schedules in alignment with the new, co-created criteria prior to and during the intervention. During the intervention, there was a 4-percentage point decrease in work schedules that did not meet any criteria, compared to before the intervention. Conversely, there was a 6-percentage point increase in work schedules meeting one criterion, but a slight 1.9-percentage point decrease in those meeting both criteria. However, none of these changes exhibited statistical significance (*p* > 0.05).

**Table 4:** Work schedules meeting none, one or both criteria weekly prior to and during the intervention

	Prior to intervention			During intervention				
**Work Schedules meeting**	Week 1	Week 2	**Average**	Week 1	Week 2	Week 3	Week 4	**Average**
**No criteria (%)**	32.8	20.8	**26.2**	23.4	17.2	22.4	26.3	**22.2**
**One criteria (%)**	14.1	15.6	**14.9**	15.6	28.7	21.1	17.1	**20.9**
**Both criteria (%)**	53.1	63.6	**58.9**	61.0	54.0	56.6	56.6	**57.0**

The criteria were 1) no more than three category 4 citizens per work schedule, and 2) no category 4 citizens in a row

### Psychosocial work demands, fatigue and exertion

The assessment of psychosocial work demands, fatigue and exertion prior to and after the intervention is presented in Table 5 showing a reduction for all parameters at follow up. Physical and emotional fatigue and physical exertion showed statistical significant change (*p* < 0.05), with a mean difference of 17 (CI = 3.67–30.70) and 11 (CI = 2.56–19.32) and 1.7 (CI = 0.29–3.08) points respectively.

**Table 5:** Psychosocial work demands, fatigue and exertion at baseline and during the intervention (follow-up)

	Baseline (*n* = 19)	Follow-up (*n* = 16)	Baseline ÷ Follow up
	Mean (SD)	Mean (SD)	Mean dif. (95% CI)
**Psychosocial work demands (100 point scale)**			
Workload	62 (12)	59 (18)	4 (−3.87, 11.68)
Work pace	57 (18)	49 (13)	7 (−2.87, 16.44)
Emotional demands	40 (18)	39 (18)	3 (−7.09, 12.29)
Influence at work**	56 (11)	61 (18)	−5 (−13.70, 3.54)
**Fatigue (100 point scale)**			
Physical	63 (22)	48 (19)	17 (3.67, 30.70)*
Emotional	67 (22)	53 (18)	11 (2.56, 19.32)*
**Exertion (10 point scale)**			
Physical	7.1 (1.4)	5.4 (2.7)	1.7 (0.29, 3.08)*

*SD *Standard deviation

*p-value < 0.05

**Higher score indicates more influence at work

### Physical behavior

**Fig. 4:**
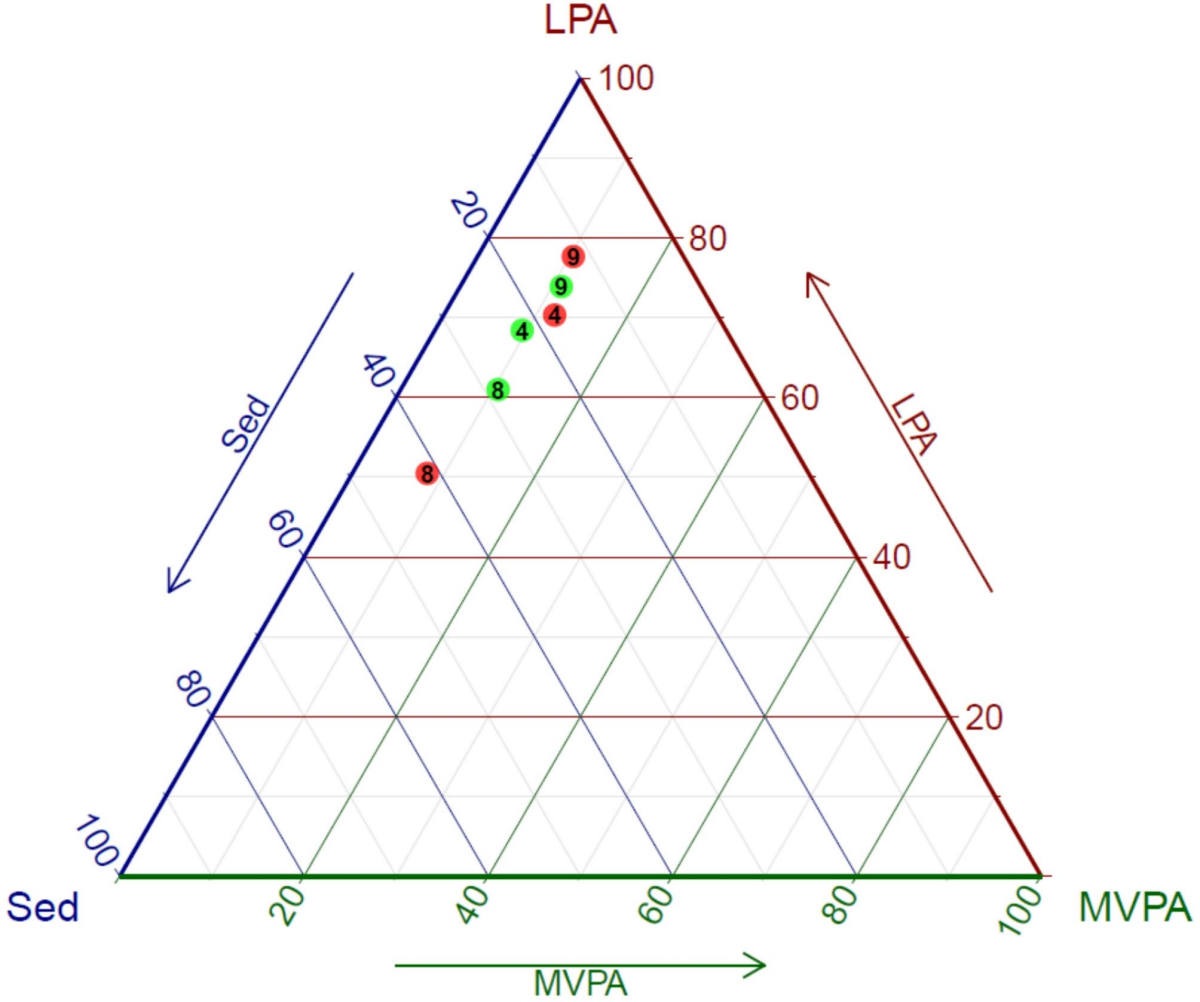
Example of the individual changes based on three HHC-aides. Average composition of a workday for three selected HHC-aides represented by ID-numbers (4, 8 and 9) at baseline (red dots) and follow-up (green dots), from the same work team. The change in individual composition (Sed, LPA, MVPA) is seen by the change in placement from the red to the green dot, e.g. ID 8 changing from a composition of 41%, 51% and 8% at baseline to 28%, 61% and 11% at follow-up. Sed: Sedentary (sitting and lying), LPA: Light physical activity (standing, standing with movement, walking slow), MVPA: Moderate to vigorous activity (walking fast, running, stair climbing, cycling)

Physical behavior measured with accelerometer was collected for 15 HHC-aides at baseline and 14 HHC-aides at follow-up. Physical behavior on group level at baseline and follow-up are presented in Table 6 for each behavior, showing no significant change between the pre and post measurements. The composition of the workday was divided into Sedentary (Sed), Light Physical Activity (LPA) and Moderate to Vigorous Activity (MVPA), resulting in an average composition of 26.1%, 63.4% and 10.7% for baseline respectively, and 24.2%, 64.2% and 11.7% for follow-up respectively. During an average workday of 7.16 and 7.03 h for baseline and follow up respectively, the main activity was standing, with 37.1% at baseline and 37.0% at follow-up. To present an example of the individual changes in composition of the workday a ternary plot with selected participants is presented in Fig. 4. The figure shows a tendency towards active participants being less physical active, and less physical active participants being more active at follow-up compared to baseline. A complete figure with all participants, divided by teams, are presented in the Appendix C.

**Table 6:** Physical behavior for an average workday presented on group level

	Baseline (*n* = 15)		Follow-up (*n* = 14)		Baseline ÷ Follow-up (*n* = 14)	
	**Mean %**	**(SD)**	**Mean %**	**(SD)**	**Mean dif. %**	**(95% CI)**
Sitting	25.7	(8.4)	23.9	(6.0)	0.09	(−0.42, 0.60)
Standing	37.1	(7.4)	37.0	(5.7)	0.12	(−0.29, 0.53)
Standing with movement	15.9	(3.2)	17.3	(4.4)	−0.08	(−0.27, 0.10)
Walking slow	10.3	(4.0)	10.0	(3.4)	0.04	(−0.16, 0.24)
Walking fast	2.5	(2.8)	2.8	(3.9)	0.00	(0.15, 0.16)
Running	0.0	(0.0)	0.0	(0.0)	0.00	(0.00, 0.00)
Stair climbing	1.8	(0.7)	2.3	(1.9)	−0.04	(−0.11, 0.03)
Cycling	6.7	(2.2)	6.7	(1.8)	0.02	(−0.13, 0.17)

Group mean presented as percentage of the mean total duration with standard deviation (SD) and *p*-value with 95% CI for the differences between baseline and follow-up measures

## Discussion

The findings from the study provide valuable insights into the feasibility of an organizational intervention aimed at achieving a more equitable distribution of physical and psychosocial work demands among HHC aides. While certain components of the intervention were deemed feasible, others revealed barriers that require adaptations to enhance feasibility. This discussion will elaborate on the implications of these findings, focusing on the acceptability of intervention activities, fidelity to the intervention model, changes in psychosocial work demands and physical exertion, practical implications for the HHC sector, as well as the strengths and limitations of the study.

### Acceptability of intervention activities

One of the key aspects of this study was the acceptance of the intervention activities among HHC aides. The generally positive response of the intervention indicates a willingness among workers to engage with new scheduling practices aimed at balancing work demands. However, they did not perceive any effect of the intervention and emphasized time and complexity as work-task related factors conflicting with the intervention activities. In a similar study by Czuba et al., they reported that “*most health care aides favored limiting their schedule with respect to the number of physically demanding patients assigned to them in a day*”, and a Norwegian study suggests that the SC should distribute work schedules with different physical work demands more equally to improve health of HHC-workers [29]. This supports the relevance of the aim of this study. That said, the interviewees highlighted time pressure as a conflicting factor for successful implementation, which is known as a considerable challenge in general in the Danish home care system [30]. A study by Clausen et al. revealed that ‘more time to meet the need of the users’ and ‘regulation of work pace’ were among the most frequently cited job changes that could have caused HHC-workers to stay in their job for workers who either quit or retired [31]. Thus, for successful implementation, adaptions are needed to ensure that the intervention activities of this study fit the timeframe, not challenge it.

### Fidelity

Fidelity to the intervention was a critical area of concern, as the study found no noteworthy difference between the proportion of work schedules meeting the new criteria prior to and during the intervention. This could be due to missing classification of citizens, which was noted by HHC-aides in the interview. They indicated that citizens missing a classification would most likely be in lower categories, as HHC-aides and SC tended to be very attentive to category 4 citizens. Our results showed that the average number of planned visits in homes of category 4 citizens per day were under three both prior to and during the intervention, indicating that the threshold of three set in the criterion is feasible. However, the manual entry of classifications into the digital system was a time-consuming task, leading to the incomplete integration of classifications and subsequently affecting the distribution of citizens based on their classification.

Organizational-level interventions often have lower implementation rates compared to individual-level interventions [30, 32]. Organizational interventions involve changing workplace structures and require involvement from multiple individuals. In contrast, individual-level interventions primarily involve the individual. Participating in the classification workshop and engaging in dialogue with SC required minor adjustments to HHC-aides’ daily routine. However, planning schedules that adhere to the criteria necessitated changes to the planning system and cooperation among management, SC, and HHC-aides. To improve feasibility in future studies, we suggest automating the integration of classifications into the digital planning system. This requires a thorough understanding of the planning system and early involvement of the SC. However, time and resource limitations hindered completion of these activities in this study. A well-planned strategy is necessary for effective implementation. Czuba et al. suggests a phased implementation strategy, which can allow for more time and minimize disruption to established relationships between HHC-aides and citizens [2].

### Change in psychosocial work demands, fatigue and exertion

In terms of changes in psychosocial work demands, fatigue, and physical exertion, the study found statistically significant reductions in both physical and emotional fatigue. However, as the study design did not include a control group, we cannot solely conclude that these changes occurred based on the intervention. The observed mean difference in perceived physical exertion suggests that while the intervention may have had a positive impact, high levels of exertion still persist among HHC aides, as both baseline and follow up measures was above 4 points, a score found to indicate that high muscular loading occurs [33]. This suggest room for improvement of the intervention and/or implementation in the future.

### Practical implications

HHC-aides confirmed the necessity of interventions targeting work task distribution, providing further justification for enhancing the current intervention. HHC-aides expressed acceptance of citizen classification and reorganized work schedule criteria, which could serve as valuable tools for both SC and self-coordinating teams. The latter is a growing trend in the HHC-sector, where supportive tools are crucial for ensuring a safe work environment. Currently, citizen characteristics such as physical and psychosocial capacity are not considered when assigning work schedules, leaving HHC-aides vulnerable to physical and mental strain caused by overloaded schedules with high-dependency citizens. This issue is likely to persist even with changes in organizational structure, such as transitioning from central coordination to self-coordination models like the Buurtzog model [34]. Thus, future studies should investigate both implementation and effect of the intervention on physical and mental health in both central coordinating and self-coordinating HHC-institutions.

### Strengths and limitations

One strength of this study was the participatory approach, involving HHC-aides in designing and delivering the intervention, tailored to the specific HHC-institution. The process followed evidence-based approaches and best practices in intervention development [17]. Another strength was the use of a multiple methods, using qualitative and quantitative data to provide a fuller picture that can enhance understanding of the development and implementation of the intervention [35], including validated wearable sensors for a reliable physical activity assessment [25].

However, there are limitations to consider. The study had a small population, limiting generalizability, due to resource constraints and specific eligibility criteria, being restricted to HHC-aides only. The short intervention period may have restricted the opportunity for substantial changes to be observed. During the development phase, researchers actively engaged with HHC-aides, which could have influenced their responses in favor of the intervention and introduced bias in the results. Additionally, the study lacked a control group, making it difficult to attribute observed changes solely to the intervention. This absence raises the possibility of a Hawthorne effect, where participants may alter their behavior simply because they are being observed [36]. To address this limitation, this study could have included a pre-post evaluation of another home care unit within the same home care organization.

## Conclusion

In conclusion, this study provides insight into the feasibility of an organizational intervention aiming to redistribute physical and psychosocial work demands more equally between HHC-workers. The study found components of the intervention to be feasible, but concludes that adaptions to enhance implementation addressing barriers related to time pressure, improving fidelity to the intervention, and ensuring practical applicability within the HCC context are critical for future success. Specifically, we recommend; allowing more time for dialogue with SC and the classification workshop; gaining further insight into the digital planning system and allocating time for implementing changes to the system; develop strategies to ensure the classification of all citizens, including newly enrolled citizens; and an evaluation of the intervention using a randomized controlled design to determine potential occupational health benefits and cost-effectiveness. Continued research in this area is essential to develop effective strategies that promote the well-being of HHC workers and improve the quality of care provided to citizens.

## Supplementary Information

Supplementary Material 1.

Supplementary Material 2.

Supplementary Material 3.

## Data Availability

The datasets used and/or analysed during the current study are available from the corresponding author on reasonable request.

## Abbreviations

HHC: Home health care
SC: Scheduling coordinator
LPA: Light physical activity
MVPA: Moderate to vigorous physical activity
TFA: Theoretical framework of acceptability
