# Supplementary material for: Reorganization of work schedules for better distribution of work demands in home health care – a feasibility study
Source: BMC Health Serv Res. 2025 Apr 26;25:608. doi: 10.1186/s12913-025-12746-1 (PMC12032747; doi:10.1186/s12913-025-12746-1)
Supplement: Supplementary file 2 — Supplementary Material 2. [file 12913_2025_12746_MOESM2_ESM.docx]

# **APPENDIX B**

# **Semi structured interview guide for group interview with employees investigating intervention acceptability**

| **Theme (Based on The Framework of Acceptability)** | **Main questions** | **Followup questions** |
| --- | --- | --- |
| **Introduction to the interview (briefing)** | Introduction to the interview includes:   - Information about the study (purpose and content) - Structure of the interview (purpose, focus, time, roles) - Information about voluntary participation, anonymization of participants, data protection and rights of participants. - Ask for permission to record and transcribe the interview |  |
| **Introduction of employees** | Please introduce yourself.  What is your name and seniority? |  |
| Affective attitude | How do you think it has been to be a part of thestudy? Why? |  |
|  | What did you like about the study? | Make sure to ask about each intervention component:  The classification of citizens  The dialogue with the scheduling planner  The new work schedules |
|  | What didn't you like about the study? |  |
|  | Is there anything from the study that you would like to keep or continue doing? |  |
| Burden | What has been required of you as employees to participate in the study? | How often has it been necessary to re-classify the citizens? |
|  | What would need to change for it to require less from you? |  |
| Ethicality | How do you think the project fits into the daily work and main tasks of a home healthcare assistant?  Is there anything that you had to do which you didn’t like to carry out? | Have you experienced that the project have had negative consequences for:  The teamwork  Your relations to the citizens  Your professionalism |
| Perceived effectiveness | Can you describe some changes that you have observed in your workday because of the study's activities? | It can be in relation to the classification of citizens or the dialogue with the scheduling coordinator? |
|  | Have you noticed a different distribution of citizens with the new schedules? In what way? Why or why not? | What have contributed to it? Have any of the project activities contributed to it or not? How? |
|  | What would you do if you were to plan schedules that create a better distribution of citizens and a greater balance in workload? | What would that require if it should be realized? |
| Intervention coherence | Can you describe how you believe citizen classification can contribute to a better distribution of citizens and a greater balance in workload? | Have you received sufficient information about the relationship between the project’s activities and a better distribution of workload? |
|  | Have the project activities made sense to you? The classification of citizens, the dialogue with the scheduling coordinator, and the new schedules. | What contributed to that?  What could be done in a different way? |
|  | Is there anything in the study that you don’t understand or wonder about? What? |  |
| Self-efficacy | How confident were you that you and your colleagues were able to classify the citizens in the right way? | How come do you feel like that? |
|  | Have you lacked knowledge, skills, support, or anything else to be able to participate in the activities of the project? | Did it have an impact on your perspectives on the project? |
| Opportunity costs | Is there anything that you have had to down prioritize or compromise during the study? | Why was that necessary? How could one have avoided that? |
| General acceptability | Would you like the work schedules to continuously be organized in the same way as during the study? | Why/why not?  What would be required for you to wish that? |
|  | What would you like to maintain from the study, and what would you prefer to skip or change? | Why? What difference would that do? |
| The intervention in the future | If we were to continue the study, how often do you think you should:  Classify the citizens?  Have the dialogue with the scheduling coordinator? |  |
| Debriefing of the interview | I think I have the questions needed.  Is there anything you want to comment on or ask about?  Thank you for your participation. |  |
