# Supplementary material for: Reorganization of work schedules for better distribution of work demands in home health care – a feasibility study
Source: BMC Health Serv Res. 2025 Apr 26;25:608. doi: 10.1186/s12913-025-12746-1 (PMC12032747; doi:10.1186/s12913-025-12746-1)
Supplement: Supplementary file 3 — Supplementary Material 3. [file 12913_2025_12746_MOESM3_ESM.docx]

# **APPENDIX C**


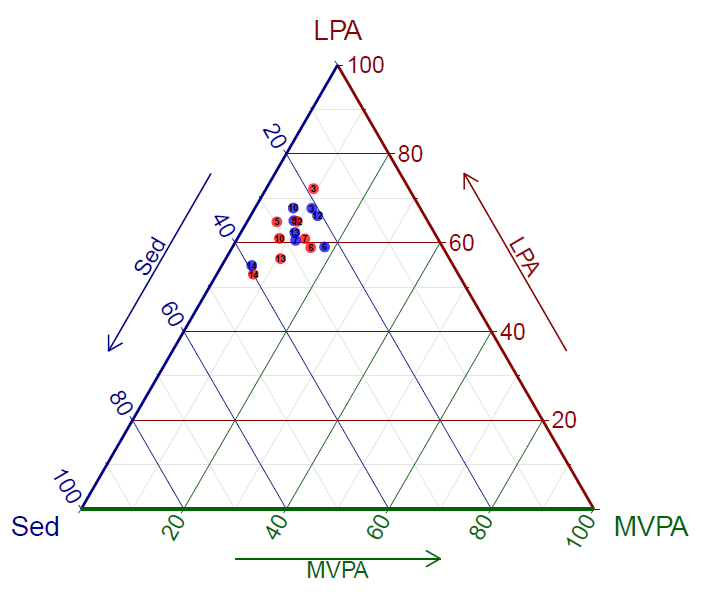

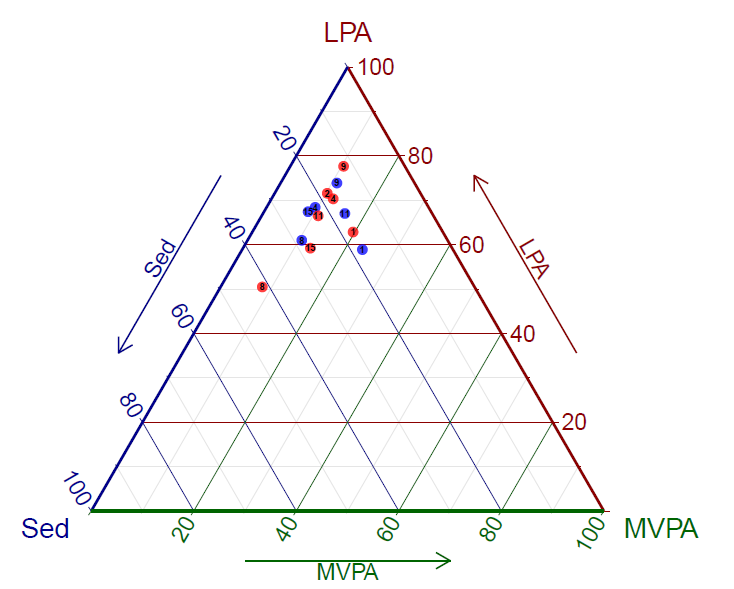


***Figure 4:*** *Average composition of a workday for each HHC-aides at baseline (red) and follow-up (blue), presented in two figures representing the two work teams (Left: Team 1, Right: Team 2). Sed: Sedentary (sitting and lying), LPA: Light physical activity (standing, standing with movement, walking slow), MVPA: Moderate to vigorous activity (walking fast, running, stair climbing, cycling).*
